# Minimally Invasive Approaches for the Management of “Difficult” Colonic Polyps

**DOI:** 10.1155/2011/682793

**Published:** 2011-06-28

**Authors:** R. Alejandro Cruz, Madhu Ragupathi, Rodrigo Pedraza, T. Bartley Pickron, Anne T. Le, Eric M. Haas

**Affiliations:** ^1^Division of Minimally Invasive Colon and Rectal Surgery, Department of Surgery, The University of Texas Medical School at Houston, 7900 Fannin, Suite 2700, Houston, TX 77054, USA; ^2^Colorectal Surgical Associates, Ltd, LLP, 7900 Fannin Street, Suite 2700, Houston, TX 77054, USA

## Abstract

Traditionally, patients with colonic polyps not amenable to endoscopic removal require open colectomy for management. We evaluated our experience with minimally invasive approaches including endoscopic mucosal resection (EMR), laparoscopic-assisted endoscopic polypectomy (LAEP), and laparoscopic-assisted colectomy (LAC). Patients referred for surgery for colonic polyps were selected for one of three minimally invasive modalities. A total of 123 patients were referred for resection of “difficult” polyps. Thirty underwent EMR, 25 underwent LAEP, and 68 underwent LAC. Of those selected to undergo EMR or LAEP, 76.4% were successfully managed without colon resection. The remaining 23.6% underwent LAC. Nine complications were encountered, including two requiring reoperative intervention. Of the 123 patients, three were found to have malignant disease on final pathology. Surgical resection can be avoided in a significant number of patients with “difficult” polyps referred for surgery by performing EMR and LAEP. In those who require surgery, minimally invasive resection can be achieved.

## 1. Introduction

First documented through sigmoidoscopy in 1968 [1], the prevalence of adenomatous polyps of the colon and rectum has been reported in up to 25% of the population [2]. Although these polyps are considered benign, their pre-neoplastic nature has been well established and removal is recommended to interrupt their malignant progression [3–6]. Selecting a suitable approach for removal may prove challenging and must be individualized with regard to the safety, feasibility, and efficacy of the modality [7]. Endoscopic polypectomy is the gold standard approach for the removal of colonic polyps [6]. However, 2–10% of lesions are considered unamenable to endoscopic removal due to technical limitations [8–10] and certain types of polyps, such as large or sessile in nature, may be associated with increased risk for colonic perforation or bleeding [9–11].

Endoscopic mucosal resection (EMR) is the least invasive alternative to standard polypectomy and is viable in the setting of large colonic polyps. EMR involves submucosal injection to lift the lesion and piecemeal removal [12, 13]. Complete removal is achieved in 83% of cases [14]. Nevertheless, EMR may prove unsuccessful when confronted with lesions situated in a tortuous colonic segment or behind a mucosal fold. When presented with these challenges, laparoscopic-assisted endoscopic polypectomy (LAEP) is preferred as this approach confers improved visualization for identification and removal of lesions [4]. The safety and efficacy of LAEP for the management of “difficult” colonic polyps has been demonstrated in several studies [4, 15–17].

Ultimately, surgical resection may be required when both EMR and LAEP fail. Open colorectal resection was the procedure of choice for lesions not amendable to endoscopic removal until 1998, when one of the first comparative studies between open and laparoscopic-assisted colectomy (LAC) showed definitive advantages for LAC with regard to earlier return of bowel function and return to normal activities [18]. Subsequently, supplementary studies have confirmed these findings and demonstrated additional positive outcomes including low conversion and complication rates, promoting LAC as the preferred approach for “difficult” colonic polyps [19–22]. We assessed our experience utilizing one of three minimally invasive surgical (MIS) modalities (EMR, LAEP, and LAC) for the treatment of large colonic polyps considered unamenable to conventional endoscopic polypectomy.

## 2. Materials and Methods

Between January 2006 and July 2010, a total of 123 consecutive patients were referred to our institution with a diagnosis of “difficult” colonic polyp for which surgical resection was initially recommended after unsuccessful attempt at endoscopic removal. “Difficult” colonic polyps included those lesions that were large or broad-based, located behind mucosal folds or in tortuous colonic segments, could not be elevated for complete removal, or were associated with increased risk for complication. Based on colonoscopy and pathology reports, patients were offered one of three MIS modalities for removal: EMR, LAEP, or LAC. The procedures were performed by one of three minimally invasive colorectal surgeons (A. T. Le, T. B. Pickron, and E. M. Haas). 

All patients were offered EMR if not previously attempted. Laparoscopic-assisted endoscopic polypectomy was offered as the procedure of choice if EMR was attempted but unsuccessful due to technical limitations (i.e., located behind mucosal folds or in tortuous colonic segments). Patients in which there was a concern for possible malignancy (i.e., failure of polyp to lift or large size) or safety (i.e., risk of perforation or bleeding) underwent LAC. Laparoscopic colorectal resection was also performed as a salvage procedure in the subset of patients in which EMR or LAEP was attempted but failed. Detailed technical descriptions for EMR [23], LAEP [24], and LAC [20] have previously been described. Each patient provided informed consent prior to performance of the selected MIS procedure. 

A deidentified retrospective database was created. Demographic information, including age, gender, and body mass index (BMI), American Society of Anesthesiologist (ASA) score, and history of prior abdominal surgery were obtained for all three groups. Intraoperative parameters, such as polyp characteristics (type, location, and size), conversions, and complications, were evaluated in all groups. In addition, estimated blood loss (EBL) and total operative time (OT) were obtained for LAEP and LAC cases. All patients undergoing EMR and LAEP were discharged on the day of the procedure. Length of hospital stay (LOS) and readmission rate were collected for the LAC group (including the group of patients that underwent LAC following failed EMR or LAEP). Thirty-day postoperative complications were noted for all three modalities. Preoperative and postoperative pathology were also reviewed for all groups. Data were analyzed as “intention to treat” groups.

## 3. Results

Between January 2006 and July 2010, a total of 123 patients with “difficult” colonic polyps were referred to our institution for surgical intervention. Fifty-seven women (46.3%) and 66 men (53.7%) with a mean age of 61.5 ± 10.8 years (range, 22–84 years), mean BMI of 30.3 ± 6.3 kg/m^2^ (range, 19.4–45.7 kg/m^2^), and median ASA of 2 (range, 1–4) underwent one of three MIS approaches for removal of colonic polyps: 30 EMR (24.4%), 25 LAEP (20.3%), and 68 LAC (55.3%). Demographic data, intraoperative parameters, and postoperative outcomes are presented as “intention to treat” groups in Tables 1 and 2. 

### 3.1. Endoscopic Mucosal Resection

**Figure 1:**
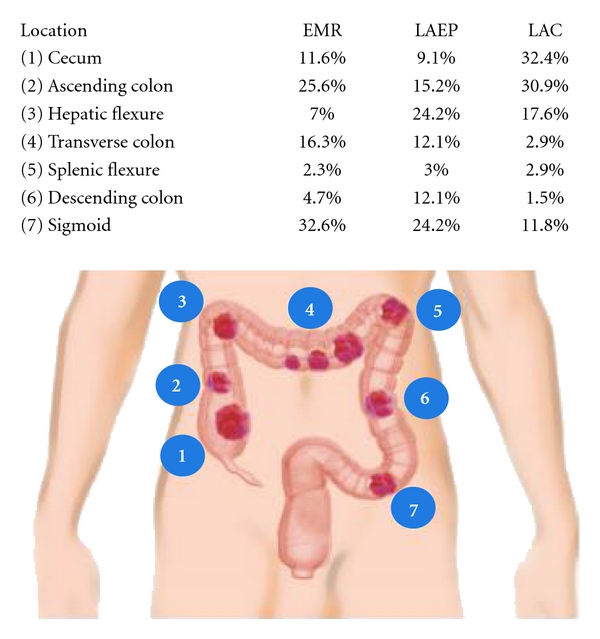
Location of polyps removed by EMR, LAEP, and LAC.

Thirty patients (12 female and 18 male) with a mean age of 61.1 ± 8.9 years (range, 47–81 years), mean BMI of 29.9 ± 5.6 kg/m^2^ (range, 21.0–38.0 kg/m^2^), and median ASA of 2 (range, 1–3) underwent EMR. The mean polyp size was 2.2 ± 0.9 cm (range, 1.0–4.0 cm). The most common location of the polyps (Figure 1) was the sigmoid colon (32.6%), followed by the ascending (25.6%) and transverse colons (16.3%). Of the 30 patients who underwent EMR, 23 patients (76.7%) had successful polyp removal. All patients were discharged on the day of the procedure, and no complications were encountered. 

Endoscopic resection was unsuccessful in 7 patients due to failed elevation with submucosal saline injection. Six of these patients required laparoscopic right hemicolectomy (RH) while one required laparoscopic anterior rectosigmoid resection (AR). The mean OT was 110.0 ± 41.8 min (range, 60–170 min), and the mean EBL was 67.9 ± 37.4 mL (range, 50–100 mL). The mean LOS was 3.2 ± 2.8 days (range, 2–4 days), and no complications were encountered. 

The majority of polyps (83.3%) removed in the EMR-treated group (including both successful and LAC-salvaged cases) were benign (Table 2). These included tubular (*n* = 16), villous (*n* = 5), and tubulovillous adenomas (*n* = 4). In addition, two serrated adenomas and one hyperplastic polyp were removed. Invasive adenocarcinoma (stage: T1N1M0) was identified in one patient necessitating a laparoscopic oncologic (anterior rectosigmoid) resection.

### 3.2. Laparoscopic-Assisted Endoscopic Polypectomy

Twenty-five patients (12 female and 13 male) with a mean age of 56.0 ± 13.8 years (range, 22–81 years), mean BMI of 29.3 ± 4.8 kg/m^2^ (range, 21.0–37.0 kg/m^2^), and median ASA of 2 (range, 1–4) underwent LAEP. Eleven patients (44.0%) had a history of prior abdominal surgery. The mean polyp size was 2.4 ± 0.9 cm (range, 1.0–4.0 cm). The most common locations of the polyps (Figure 1) were the hepatic flexure (24.2%) and sigmoid colon (24.2%). 

Nineteen patients (76.0%) were successfully treated through LAEP. The mean OT was 92.7 ± 31.0 minutes (range, 60–145 minutes), and the mean EBL was 20.0 ± 23.8 mL (range, 10–100 mL). The mean LOS was 1.5 ± 0.8 days (range, 0–2 day). Two complications, postoperative ileus and an abdominal abscess, were encountered and resolved with conservative management (nasogastric tube (NGT) decompression and antibiotics, resp.). 

Laparoscopic-assisted polypectomy was unsuccessful due to failed elevation with submucosal saline injection in 6 patients. Three of these patients underwent laparoscopic RH, two proceeded to laparoscopic transverse colectomy (TC), and a laparoscopic left colectomy (LC) was carried out in the remaining patient. The mean OT was 163.6 ± 86.1 min (range, 60–272 min), and the mean EBL was 182.5 ± 90.7 mL (range, 50–250 mL). The mean LOS was 3.5 ± 1.0 days (range, 3–5 days), and no complications were encountered. 

The majority of specimens (72.0%) removed in the LAEP-treated group (including both successful and LAC-salvaged cases) were benign (Table 2). These included tubular (*n* = 10), villous (*n* = 5), and tubulovillous (*n* = 3) adenomas. Adenocarcinoma (stage: T1N0M0) was noted in one patient, and a laparoscopic oncologic RH was subsequently performed.

### 3.3. Laparoscopic-Assisted Colectomy

Sixty-eight patients (33 female and 35 male) with a mean age of 63.8 ± 9.6 years (range, 22–81 years), mean BMI of 29.8 ± 6.8 kg/m^2^ (range, 21.0–45.7 kg/m^2^), and median ASA of 2 (range, 1–4) underwent immediate laparoscopic surgical resection. Thirty-six patients (52.9%) had prior abdominal surgery. The mean polyp size was 2.9 ± 1.2 cm (range, 1.0–8.0 cm). The most common location of the polyps (Figure 1) was the cecum (32.4%), followed by the ascending colon (30.9%) and hepatic flexure (17.6%). 

Fifty-seven RH, 7 AR, 2 TC, and 2 laparoscopic-assisted left hemicolectomies (LH) were performed. The mean OT was 119.2 ± 50.1 min (range, 31–331 min), and the mean EBL was 70.0 ± 41.2 mL (range, 25–200 mL) for all LAC procedures. No intraoperative complications were encountered, and a minimally invasive approach was preserved for all patients. Tubular adenoma was the most common histopathologic finding (Table 2). One patient that underwent a laparoscopic RH was noted to have invasive adenocarcinoma (stage: T1N1M0) on postoperative pathology.

The mean LOS was 3.5 ± 1.6 days (range, 2–8 days). Seven complications were encountered during the postoperative period. Three patients developed postoperative ileus requiring NGT decompression, two developed wound infections requiring antibiotics, and two were readmitted for secondary intervention due to development of anastomotic leaks during 30-day followup. The first patient with a leak underwent an open ileocolic resection with ileostomy while the second patient underwent a hand-assisted laparoscopic segmental resection with colostomy. No intraoperative complications were encountered, and the patients were discharged after 3 and 8 days, respectively.

## 4. Discussion

Conventional polypectomy techniques during colonoscopy are typically sufficient to remove the majority of colonic polyps; however, alternative approaches may be required when “difficult” polyps are encountered. Such lesions are characterized as large or broad-based, located behind mucosal folds or in tortuous colonic segments, incapable of being elevated for complete removal, or associated with increased risk for complication [9–11, 14, 25, 26]. We provide several alternative minimally invasive approaches (EMR, LAEP, and LAC) for patients referred for surgical resection of large colonic polyps in an attempt to avoid open colon resection. 

Endoscopic submucosal resection is associated with success rates up to 83% [27] and complication rates as low as 1.7% [13]. Although relatively successful with minimal risk for complication, there are cases where EMR is attempted but unsuccessful due to technical limitations. In our series, polyp removal was successful in 76.7% of patients, which is comparable to the success rates of 74.1% and 83% reported by Church and Lipof et al., respectively [9, 27]. Locations inaccessible by endoscopy and large or flat polyps were the most common reasons for failed EMR. In our series, no complications such as bleeding or perforation [9, 11, 25] were encountered among the patients that underwent EMR.

Laparoscopic-assisted endoscopic polypectomy was first introduced and described in 1993 by Beck and Karulf [28]. The technique allows the surgeon to control and maneuver the colon to improve visualization and access for complete polyp removal. Ideally, carbon dioxide (CO_2_) insufflation should be employed for colonoscopy as the CO_2_ readily dissolves through the tissue planes and minimizes overdistention of the colon. Manipulation through laparoscopic technique helps overcome certain technical limitations through repositioning of the colonic segment to yield an optimal relationship between the polyp and endoscopic instrument. Laparoscopic technique is utilized to release attachments (e.g., ileocolic or gastrocolic) or adhesions to assist in reorientation of the specific segment of the colon. Furthermore, applying two points of fixation using graspers can facilitate straightening and rotation of the desired colonic segment. LAEP also allows external visualization to help determine any areas of concern for immediate or delayed perforation and reinforce such areas through direct suture oversew techniques [4, 15, 16]. 

Franklin Jr. and Portillo evaluated the long-term followup and oncologic safety of LAEP in 176 patients. In their series, four patients required formal colon resection due to failure of the procedure. There were no reports of major complications or cancer recurrences [15]. They concluded that LAEP was a safe and effective MIS alternative to formal colon resections. In our series, we limited the use of LAEP to those patients in which EMR was unsuccessful, thus providing another MIS alterative to avoid formal surgical resection. The mean OT for LAEP was 92.7 ± 31.0 minutes which is similar to previously published OT of 96.5 and 100 minutes [4, 15]. Two patients experienced postoperative complications (ileus and abdominal abscess) yet there were no readmissions or reoperative interventions. Our complication rate of 2.9% is comparable to the 3% reported by Wilhelm et al. [4]. 

In cases when EMR or LAEP could not be performed (i.e., 13 failed cases), we maintained a minimally invasive approach by offering laparoscopic-assisted colectomy. A total of 81 patients (65.8%) underwent LAC as a definitive treatment for large colonic polyps. Among the patients that were offered EMR and LAEP (*n* = 55), we were able to avoid formal colorectal resection in 76.4% (*n* = 42) of patients who were initially referred for surgical resection. The benefits of LAC versus open colectomy have been well described in regard to early return of bowel function, earlier tolerance of oral intake, decreased narcotic use, reduced length of hospitalization, and overall lower complication rates [20–22, 29]. There were a total of seven complications (10.3%), which is comparable to previous published data on LAC for colon polyps ranging from 9.3% [22] to 17.7% [20].

## 5. Conclusion

Endoscopic mucosal resection, laparoscopic-assisted endoscopic polypectomy, and laparoscopic-assisted colectomy are safe and feasible in the management of “difficult” colonic polyps. Open segmental resection can be avoided in a significant number of patients referred for surgical intervention of these lesions. For patients in which EMR and LAEP is unsuccessful, a minimally invasive approach can be maintained with a laparoscopic colon resection.

## Figures and Tables

**Table 1:** Demographic data, intraoperative parameters, and postoperative outcomes for “intention to treat” groups undergoing EMR, LAEP, and LAC.

Parameter	EMR (*n* = 30)	LAEP (*n* = 25)	LAC (*n* = 68)
Gender (female : male)	12 : 18	12 : 13	33 : 35
Age (years)	61.1 ± 8.9	56.0 ± 13.8	63.8 ± 9.6
BMI (kg/m^2^)	29.9 ± 5.6	29.3 ± 4.8	29.8 ± 6.8
ASA* (range)	2 (1–3)	2 (1–4)	2 (1–4)
Past surgical history**	N/A	11 (44.0)	36 (52.9)
Success rate (%)	76.7	76.0	100.0
Operative time (min)	N/A	92.7 ± 31.0	119.2 ± 50.1
Estimated blood loss (mL)	N/A	20.0 ± 23.8	70.0 ± 41.2
Length of stay (days)	D/C on DOP	1.5 ± 0.8	3.5 ± 1.6
Postoperative complications	None	Postoperative ileus (*n* = 1)	Postoperative ileus (*n* = 3)
		Abdominal abscess (*n* = 1)	Wound infection (*n* = 2)
			Anastomotic leak (*n* = 2)

Data presented as mean ± standard deviation unless otherwise specified.

*Data for ASA presented as median (range), **data represents number (*n*) and percentage (%).

ASA: American Society of Anesthesiologists score, BMI: body mass index, D/C on DOP: discharged on date of procedure, EMR: endoscopic mucosal resection, LAC: laparoscopic-assisted colectomy, LAEP: laparoscopic-assisted endoscopic polypectomy, and N/A: not available.

**Table 2:** Characteristics and pathology of polyps removed by EMR, LAEP, and LAC.

Characteristic	EMR (*n* = 30)	LAEP (*n* = 25)	LAC (*n* = 68)
Polyp size(cm)*	2.2 ± 0.9	2.4 ± 0.9	2.9 ± 1.2

Location of polyps (%)	Sigmoid colon (32.6)	Hepatic flexure (24.2)	Cecum (32.4)
	Ascending colon (25.6)	Sigmoid colon (24.2)	Ascending colon (30.9)
	Transverse colon (16.3)	Ascending colon (15.2)	Hepatic flexure (17.6)

Pathology (%)^†^	Tubular (53.3)	Tubular (40.0)	Tubular (38.2)
	Villous (16.7)	Villous (20.0)	Tubulovillous (35.3)
	Tubulovillous (13.3)	Tubulovillous (12.0)	Villous (13.2)
	Serrated adenoma (10.0)	Adenocarcinoma (12.0)	Serrated adenoma (5.9)
	Adenocarcinoma (3.3)	Serrated adenoma (8.0)	Submucosal lipoma (4.4)
	Hyperplastic (3.3)	Hyperplastic (8.0)	Adenocarcinoma (1.5)
			Hyperplastic (1.5)

*Data provided as mean ±standard deviation.

**^†^**Includes cases salvaged by LAC (i.e., failed initial attempt at removal by EMR or LAEP).

EMR: endoscopic mucosal resection, LAC: laparoscopic-assisted colectomy, and LAEP: laparoscopic-assisted endoscopic polypectomy.

## References

[1] Overholt BF (1968). Clinical experience with the fibersigmoidoscope. *Gastrointestinal Endoscopy*.

[2] Giacosa A, Frascio F, Munizzi F (2004). Epidemiology of colorectal polyps. *Techniques in Coloproctology*.

[3] Winawer SJ, Zauber AG, May Nah Ho (1993). Prevention of colorectal cancer by colonoscopic polypectomy. The National Polyp Study Workgroup. *New England Journal of Medicine*.

[4] Wilhelm D, Von Delius S, Weber L (2009). Combined laparoscopic-endoscopic resections of colorectal polyps: 10-Year experience and follow-up. *Surgical Endoscopy*.

[5] Robertson DJ (2010). Colonoscopy for colorectal cancer prevention: is it fulfilling the promise?. *Gastrointestinal Endoscopy*.

[6] Repici A, Tricerri R (2004). Endoscopic polypectomy: techniques, complications and follow-up. *Techniques in Coloproctology*.

[7] Senagore AJ, Duepree HJ, Delaney CP, Brady KM, Fazio VW (2003). Results of a standardized technique and postoperative care plan for laparoscopic sigmoid colectomy: a 30-month experience. *Diseases of the Colon and Rectum*.

[8] Delaney CP, Champagne BJ, Marks JM, Sanuk L, Ermlich B, Chak A (2010). Tissue apposition system: new technology to minimize surgery for endoscopically unresectable colonic polyps. *Surgical Endoscopy*.

[9] Church JM (2003). Avoiding surgery in patients with colorectal polyps. *Diseases of the Colon and Rectum*.

[10] Bergmann U, Beger HG (2003). Endoscopic mucosal resection for advanced non-polypoid colorectal adenoma and early stage carcinoma. *Surgical Endoscopy*.

[11] Vokurka J (1999). Laparoscopically-assisted endoscopic polypectomy. *Rozhledy Chirurgii*.

[12] Waye JD (2001). Endoscopic mucosal resection of colon polyps. *Gastrointestinal Endoscopy Clinics of North America*.

[13] Katsinelos P, Kountouras J, Paroutoglou G (2008). A comparative study of 50% dextrose and normal saline solution on their ability to create submucosal fluid cushions for endoscopic resection of sessile rectosigmoid polyps. *Gastrointestinal Endoscopy*.

[14] Iishi H, Tatsuta M, Iseki K (2000). Endoscopic piecemeal resection with submucosal saline injection of large sessile colorectal polyps. *Gastrointestinal Endoscopy*.

[15] Franklin ME, Portillo G (2009). Laparoscopic monitored colonoscopic polypectomy: long-term follow-up. *World Journal of Surgery*.

[16] Franklin ME, Leyva-Alvizo A, Abrego-Medina D (2007). Laparoscopically monitored colonoscopic polypectomy: an established form of endoluminal therapy for colorectal polyps. *Surgical Endoscopy*.

[17] Prohm P, Weber J, Bönner C (2001). Laparoscopic-assisted coloscopic polypectomy. *Diseases of the Colon and Rectum*.

[18] Joo JS, Amarnath L, Wexner SD (1998). Is laparoscopic resection of colorectal polyps beneficial?. *Surgical Endoscopy*.

[19] Young-Fadok TM (2000). Benefits of laparoscopic-assisted colectomy for colon polyps:acase-matched series. *Mayo Clinic Proceedings*.

[20] Pokala N, Delaney CP, Kiran RP, Brady K, Senagore AJ (2007). Outcome of laparoscopic colectomy for polyps not suitable for endoscopic resection. *Surgical Endoscopy*.

[21] Lo SH, Law WL (2005). Laparoscopic colorectal resection for polyps not suitable for colonoscopic removal. *Surgical Endoscopy*.

[22] Hauenschild L, Bader FG, Laubert T (2009). Laparoscopic colorectal resection for benign polyps not suitable for endoscopic polypectomy. *International Journal of Colorectal Disease*.

[23] Luigiano C, Consolo P, Scaffidi MG (2009). Endoscopic mucosal resection for large and giant sessile and flat colorectal polyps: a single-center experience with long-term follow-up. *Endoscopy*.

[24] Franklin ME (2000). Laparoscopic-assisted colonoscopic polypectomy: the Texas endosurgery institute experience. *Diseases of the Colon and Rectum*.

[25] Soetikno R, Gotoda T (2009). Con: colonoscopic resection of large neoplastic lesions is appropriate and safe. *The American Journal of Gastroenterology*.

[26] Germer CT, Ritz JP, Buhr HJ (2003). Laparoscopic colon surgery. Indications and technique. *Chirurg*.

[27] Lipof T, Bartus C, Sardella W, Johnson K, Vignati P, Cohen J (2005). Preoperative colonoscopy decreases the need for laparoscopic management of colonic polyps. *Diseases of the Colon and Rectum*.

[28] Beck DE, Karulf RE (1993). Laparoscopic-assisted full-thickness endoscopic polypectomy. *Diseases of the Colon and Rectum*.

[29] Young-Fadok TM, Soetikno R, Gotoda T (2009). Pro: a large colonic polyp is best removed by laparoscopy. *American Journal of Gastroenterology*.

